# Right Ventricular Stroke Work Index and Its Prognostic Value in a Lung Transplant Population With Group 3 Pulmonary Hypertension

**DOI:** 10.1155/joot/1628090

**Published:** 2026-10-13

**Authors:** Yann Marquet, Sébastien Jacqmin, Julien Fessler, Edouard Sage, Pierre Hammoum, Morgan Le Guen

**Affiliations:** ^1^ Groupe de Recherche Clinique en Anesthésie Réanimation Médecine Périopératoire GRC 29 ARPE Sorbonne Université Paris F-75012 France sorbonne-universites.fr; ^2^ Department of Anesthesiology and Critical Care DMU DREAM Hôpital La Pitié-Salpêtrière, APHP Paris F-75012 France; ^3^ Department of Anaesthesiology and Pain Medicine Foch Hospital University of Versailles Saint Quentin Suresnes France; ^4^ Department of Anesthesiology Weill Cornell Medical College New York New York USA cornell.edu; ^5^ Department of Thoracic Surgery and Lung Transplantation Foch Hospital Suresnes France

**Keywords:** extracorporeal membrane oxygenation, lung transplant, pulmonary graft dysfunction, pulmonary hypertension, right ventricular stroke work index

## Abstract

**Background:**

Lung transplantation requires meticulous preoperative assessment. Recipients with Group 3 pulmonary hypertension can be at higher risk of mortality and primary graft dysfunction (PGD). We conducted a study to determine whether the right ventricular stroke work index (RVSWI), a right‐heart catheterisation haemodynamic indicator assessing right ventricle function, could predict 1‐year survival in this setting.

**Methods:**

Monocentric, retrospective, observational cohort study approved by our local ethics review board, between January 2013 and December 2023. We included lung transplant recipients who underwent preprocedural right‐heart catheterisation assessment and presented with Group 3 pulmonary hypertension. We first determined a clinically relevant RVSWI threshold. The primary endpoint was mortality at 1 year amongst two groups of patients according to this threshold.

**Results:**

66 patients (age 57.0 ± 11.4 years, male 56%) were included amongst a study population of 699 patients. Double‐lung transplantations represented most of the procedures (91%). In univariate analysis, RVSWI was statistically associated with PGD Grade 3 at Day 3 and intraoperative veno‐arterial extracorporeal membrane oxygenation (ECMO) at a threshold of RVSWI = 13.5. Mortality at 1 year did not differ between the groups RVSWI ≤ 13.5 (6 patients, 14.6%) and RVSWI > 13.5 (6 patients, 24%) even after accounting for potential confounding factors.

**Conclusions:**

In our lung transplant population with Group 3 pulmonary hypertension, RVSWI was not associated with mortality at 1 year. A higher RVSWI seemed associated with significant events such as intraoperative ECMO and PGD Grade 3. Further studies are needed to assess RVSWI’s prognostic value in this setting.

## 1. Introduction

Lung transplantation (Ltx) is the treatment for end‐stage lung disease. Nevertheless, it bears significant morbidity and mortality rates owing to recipient, donor and technical factors, and thus requires meticulous preprocedural assessment. Primary graft dysfunction (PGD) is a significant syndrome of acute lung injury occurring in 15%–30% of the patients during the first 72 h following Ltx [1–3]. Severe PGD at Day 3 is associated not only to early and late mortality but also to chronic complications [2, 4, 5]. Moreover, preoperative pulmonary hypertension (PH) is associated to worse outcomes in lung transplant recipients, especially mortality [2, 6]. Although conflicting results have emerged, it is also considered to be a recipient‐related risk factor for PGD [1, 7–10].

Cardiac assessment is paramount in Ltx setting, and various modalities are possible [11, 12]. Right‐heart catheterisation (RHC), although invasive, allows a comprehensive haemodynamic assessment. Right ventricular stroke work index (RVSWI) is a surrogate for the right ventricle (RV) workload and contractility capacity, reflecting its ability to pump blood against resistance. It is calculated using measurements obtained during RHC: It has been associated with postoperative right‐heart failure after left ventricle assistance device surgery and is gaining attention in several settings including PH and heart failure. Low RVSWI values (i.e., under 6 g m m^−2^ beat^−1^ in a population without PH) in these settings are associated with poor outcomes, especially acute right‐heart failures, hospitalisations and mortality [13–16]. On the other hand, RVSWI has previously been investigated in a single Ltx retrospective group of patients and showed a pejorative influence on mortality when it was not altered [17]. There is an apparent discrepancy between these results and a knowledge gap on the matter. Nonetheless, RVSWI could be a useful index when applied to lung transplant candidates with PH as it might reflect the RV adaptation to chronic lung diseases and their related changes in pulmonary vasculature. Therefore, it might indirectly integrate dynamic features such as pulmonary vascular compliance and RV–arterial coupling. To our knowledge, there are no reports on RVSWI evaluation in Ltx recipients with Group 3 PH.

We sought to investigate the prognostic value of RVSWI in an Ltx population with Group 3 PH. We first tried to determine a specific RVSWI threshold to split our population into two groups with “high” and “low” values. We hypothesised that in our study population, a high RVSWI would lead to an increase in mortality. We further hypothesised that PGD incidence would differ amongst low RVSWI and high RVSWI populations.

## 2. Material and Methods

We retrospectively investigated adult patients who underwent an Ltx in a French tertiary care hospital from January 2013 to December 2023. We assessed for eligibility patients who had a preoperative history of PH. Patients were included if they went through RHC with haemodynamic assessment less than 12 months before surgery and had a concomitant diagnosis of isolated precapillary or combined pre‐ and postcapillary PH. We did not include patients who underwent multiorgan transplantation (i.e., other organ transplantation on the same surgical procedure), redo transplant procedure or when cardiopulmonary bypass was used during surgery. Patients who lacked data to properly assess mortality up to 1 year and PGD grading were excluded.

This study followed the Strengthening the Reporting of Observational Studies in Epidemiology (STROBE) reporting guideline.

### 2.1. Ethical Considerations

This study was conducted in accordance with French laws pertaining to observational, noninterventional and retrospective analyses of medical records, and the study was approved by the ethics review board of Foch Hospital (IRB 00012437), number of approval: 24‐07‐13.

### 2.2. RHC and Haemodynamic Assessment

RHC parameters of interest were cardiac output, cardiac index (CI), pulmonary pressures including mean pulmonary arterial pressure (mPAP), pulmonary arterial wedge pressure (PAWP), mean right atrial pressure (mRAP) and pulmonary vascular resistance (PVR). Stroke volume index (SVI) and RVSWI were calculated from these parameters using the following equations:

• SVI (mL beat^−1^ m^−2^) = CI (L minute^−1^ m^−2^) × 1000/heart rate (beat minute^−1^)
• RVSWI = ((mPAP)‐(mRAP)) × SVI × 0.0136, where RVSWI is expressed as gram‐force metre per beat and is indexed to body surface (g m m^−2^ beat^−1^).

PH was diagnosed according to the latest recommendations [18]: PH was diagnosed if mPAP was > 20 mmHg; it was deemed precapillary when associated with both PAWP ≤ 15 mmHg and PVR > 2 WU and was deemed combined pre‐ and postcapillary when associated with both PAWP > 15 mmHg and PVR > 2 WU. We collected concurrent echocardiographic and cardiac resonance imaging data whenever they were performed.

### 2.3. Ltx Procedure

Surgery consists in a sequential Ltx involving one or two anterolateral thoracotomies. The standardised perioperative anaesthetic management protocol used in our institution has been published elsewhere [19]. Intraoperative haemodynamic monitoring includes systemic arterial pressure continuous monitoring, pulmonary artery catheterisation and transesophageal echocardiography (TEE). A standardised TEE examination is performed at each step of the procedure. In cases of fibrosis‐related pathologies, our local management includes veno‐arterial (VA) extracorporeal membrane oxygenation (ECMO) implementation after dissection of the first native lung as a lung‐protective strategy: the goal is to relieve haemodynamic and ventilatory stress on grafts for up to 4 days. High emergency lung transplantation (HELT) allocation is available in our country for candidates with severely impaired respiratory status [20].

### 2.4. PGD Grading

PGD was assessed according to the 2016 ISHLT definition [4]. Namely, PGD was assessed at T0, T24, T48 and T72, where T0 is the time of reperfusion of the second lung (or first lung in single Ltx). PaO_2_/FiO_2_ ratio was recorded at these times for each patient, and whenever it had not been available within 6 h interval, SpO_2_/FiO_2_ ratio was used and the cutoff values for PGD severity were changed accordingly. We reported the worst PaO_2_/FiO_2_ ratio when several arterial blood gases values were available for a given calendar. Use of ECMO was reported and PGD was graded accordingly by consensus by a board‐certified panel including an intensivist, a pulmonologist and an anaesthesiologist: either Grade 3 if bilateral pulmonary oedema compatible with PGD was present on the chest X‐ray or ungradable.

### 2.5. Outcome Measures, Data Assessment and Statistical Analyses

As no previous report exists on the prognostic impact of RVSWI in Ltx for patients with Group 3 PH, we first sought to ascertain the association of RVSWI with several remarkable events with receiver operating characteristic (ROC) curve analyses and extractions of Youden points. The events included 1‐month mortality, 1‐year mortality, Grade 3 PGD at Day 3 (PGD 3‐T72) and intraoperative use of ECMO. The most relevant cutoff value from these analyses was used to split our population for subsequent comparisons and analyses. The primary endpoint was 1‐year mortality. The secondary endpoints included 1‐month mortality, PGD 3‐T72 and intraoperative ECMO requirement.

Data were extracted from the electronic medical records.

Results for categorical variables, expressed as number (%), were compared using the *χ* ^2^ test or Fisher’s exact test when appropriate. Continuous variables, expressed as mean (± standard deviation) or median (25th–75th percentile interquartile range [IQR]), were compared using Student’s *t*‐test or Wilcoxon’s rank‐sum test, depending on data distribution. The distribution of continuous variables was evaluated using graphical data visualisation techniques.

To determine the optimal threshold value for RVSWI, a ROC curve analysis was performed. The Youden index was applied to identify the optimal cutoff point for RVSWI, which was subsequently used to stratify patients. Kaplan–Meier survival curves were constructed to compare outcomes between the groups defined by this threshold, and the log‐rank test was used for statistical comparison.

The primary endpoint was assessed using logistic regression analysis. Initially, a bivariate analysis was conducted to explore associations between clinical variables and the primary outcome. Variables with clinical relevance or statistical significance in bivariate analysis were included in a multivariate logistic regression model adjusted for key baseline covariates associated with early mortality in the literature, including double‐lung transplant, ECMO use during surgery, HELT procedure, PGD 3‐T72 and mPAP.

Secondary endpoints were assessed using bivariate analyses without multivariate adjustment.

A sensitivity analysis of the primary endpoint was performed using the same methodology, applying an alternative RVSWI threshold defined as the 75th percentile of RVSWI in our study population.

All statistical analyses were performed using *R* Studios Version 2025.05.0 + 496 (2025.05.0 + 496) and *R* 4.5.0. A two‐sided *p* value < 0.05 was considered statistically significant.

## 3. Results

### 3.1. Characteristics and Main Transplantation‐Related Outcomes

From January 2013 to December 2023, 699 patients underwent Ltx in our institution and were retrospectively assessed. There were 218 patients with a past medical history of PH. Amongst them, 75 had a recent preoperative RHC assessment and did not benefit from CPB, redo or multiorgan transplantation. 66 patients with a PH were finally included: 63 patients who had a precapillary PH and 3 who had combined pre‐ and postcapillary on RHC assessment. No patient has been excluded as no data were missing on main outcome (Figure 1). They were mostly male (56%), with a mean age of 57.0 ± 11.4 years. The primary end‐stage lung diseases were mostly emphysema (42%) and pulmonary fibrosis (39%). The median preoperative mPAP was 30 [25–36] mmHg and median PVR was 4.6 [3.0–5.6] Wood Units. Median RVSWI was 11.2 [7.9–15.1] g m m^−2^ beat^−1^ in our population (Table 1).

**FIGURE 1:**
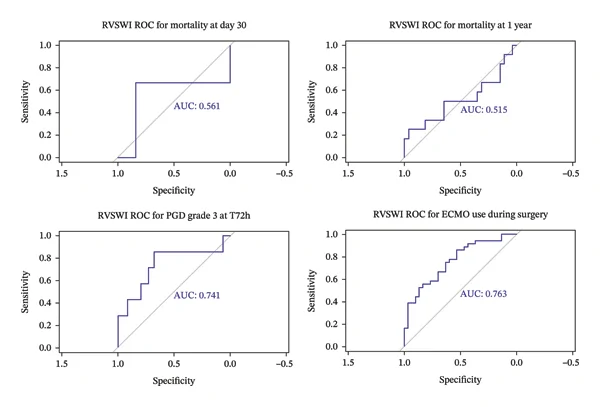
Receiver operating characteristic (ROC) curve for RVSWI with significant perioperative events. Abbreviations: RVSWI = right ventricular stroke work index, PGD = primary graft dysfunction, ECMO = extracorporeal membrane oxygenation.

**TABLE 1:** Recipient, donor and lung transplant characteristics of the study population.

Variables	*n* = 66
Recipient characteristics	
Male	37 (56)
Body mass index, kg m^−2^	23.5 ± 4.6
Age, years	57.0 ± 11.4
Pulmonary transplant type	
Single lung	6 (9)
Bilateral	60 (91)
Pulmonary disease	
Emphysema	28 (42)
Fibrosis	26 (39)
Cystic fibrosis	5 (8)
Other bronchiectasis	6 (9)
Others	1 (2)
High emergency transplantation procedure	4 (6)
Pretransplantation ECMO implementation	4 (6)
Right‐heart catheterisation measures	
mPAP, mmHg	30 (25–36)
Pcap, mmHg	10 (7–12)
Cardiac index, L min^−1^ m^−2^	2.8 (2.2–3.4)
Pulmonary vascular resistance, Wood unit	4.6 (3.0–5.6)
Right ventricular stroke work index, g m m^−2^ beat^−1^	11.2 (7.9–15.1)
Donor characteristics	
Male	32 (48)
Sex mismatch	20 (30)
Age, years	50.3 ± 17.6
Body mass index, kg m^−2^	24.6 ± 4.6
Smoking history	26 (39)
Pack year	15 (6–20)
Last known donor PaO_2_/FiO_2_ ratio	357 (315–450)
Lung transplantation procedure	
No ex vivo lung perfusion procedure	47 (71)
First lung ischemia, min	268 (229–302)
Second lung ischemia, min	401 (330–423)
Single lung ischemia (*n* = 4), min	277 (269–286)
Ex vivo lung perfusion procedure	19 (29)
Ex vivo procedure duration, min	216 (180–228)
First lung ischemia, min	623 (571–657)
Second lung ischemia, min	743 (687–832)
Single lung ischemia (*n* = 2), min	553 (504–602)
FiO_2_ first lung reperfusion	0,8 (0,6–1)
FiO_2_ second lung reperfusion	0,8 (0,55–1)
Per‐transplantation transfusion	
RBC, units	3 (2–6)
FFP, units	3 (2–6)
Platelets, units	0 (0–0)

*Note:* Continuous variables are expressed as median (interquartile range 25–75) or as mean ± standard deviation; categorical variables are expressed as No. (%).

Abbreviations: ECMO = extracorporeal membrane oxygenation, FFP = fresh frozen plasma, mPAP = mean pulmonary artery pressure, Pcap = capillary pulmonary pressure, RBC = red blood cells.

Ltx procedures were mostly bilateral (91%). Ex vivo lung perfusion was carried out in 19 procedures (29%) before transplant. VA ECMO was performed preoperatively in four patients due to acute worsening of their respiratory conditions and was subsequently registered on the high emergency graft allocation list. Including the previous patients, ECMO was performed on 36 patients during surgery, mainly with a peripheral set‐up and once with a centrally implanted ECMO. VA ECMO was implanted for PH and right‐heart failure in 23 cases (45%). ECMO was weaned at the end of the procedure (i.e., in the operating room before transfer to ICU) in 18 cases. In the other cases, median ECMO duration was 4 days [3–10]. First implemented ECMO was always VA ECMO but was secondarily switched for veno‐venous ECMO in 4 patients in the postoperative setting. ECMO procedures are detailed in Table 2. Patients spent a median of 22 [15–42] days and 43 [27–65] days, respectively, in the ICU and in the hospital before discharge. PGD 3‐T72 occurred in seven patients (11%), and three patients (5%) died within 30 days, whereas 12 died (18%) within 1 year (Table 3).

**TABLE 2:** Transplantation ECMO procedures.

Variables	*n* = 36
Peripheral VA ECMO	35 (97)
Central VA ECMO	1 (3)
VA ECMO implementation aetiology	
Pulmonary hypertension and right‐heart failure	23 (64)
Refractory hypoxemia or hypercapnia	2 (6)
Hemorrhagic shock or acute surgical complication	3 (8)
Cardiac arrest	1 (3)
Other unspecified circulatory insufficiency	1 (3)
Lung protection strategy	6 (17)
VA ECMO management	
VA ECMO preoperative implementation	4 (11)
VA ECMO intraoperative implementation	32 (89)
VA ECMO maintained postsurgery	18 (50)
VA ECMO switched to VV ECMO	4 (11)
ECMO duration, days	4 (3–10)

*Note:* Continuous variables are expressed as median (interquartile range 25–75); categorical variables are expressed as No. (%).

Abbreviations: ECMO = extracorporeal membrane oxygenation; VA = veno‐arterial; VV = veno‐venous.

**TABLE 3:** Lung transplantation outcomes of the study population.

Variables	*n* = 66
T0 PGD grading	
T0 PGD 0	24 (36)
T0 PGD 1	8 (12)
T0 PGD 2	5 (8)
T0 PGD 3	24 (36)
T0 PGD ungradable	5 (8)
T24 PGD grading	
T24 PGD 0	27 (41)
T24 PGD 1	12 (18)
T24 PGD 2	4 (6)
T24 PGD 3	14 (21)
T24 PGD ungradable	9 (14)
T48 PGD grading	
T48 PGD 0	33 (50)
T48 PGD 1	9 (14)
T48 PGD 2	6 (9)
T48 PGD 3	10 (15)
T48 PGD ungradable	8 (12)
T72 PGD grading	
T72 PGD 0	34 (52)
T72 PGD 1	8 (12)
T72 PGD 2	10 (15)
T72 PGD 3	7 (11)
T72 PGD ungradable	7 (11)
Other outcomes	
Time in ICU, days	22 (15–42)
Time in hospital, days	43 1 (27–65)
Mortality within 30 days	3 (5)
Mortality within 1 year	12 (18)

*Note:* Continuous variables are expressed as median [interquartile range 25–75]; categorical variables are expressed as No. (%). T0 = time of second lung reperfusion, T24 = 24 h post‐second lung reperfusion, T48 = 48 h post‐second lung reperfusion, T72 = 72 h post‐second lung reperfusion.

Abbreviation: PGD = primary graft dysfunction.

### 3.2. RVSWI Threshold Assessment

ROC curve analyses were computed to assess binary association between RVSWI and mortality at 1 year, mortality at 30 days, PGD 3‐T72 and intraoperative ECMO. The corresponding curves can be found on our supporting tables. RVSWI was not associated with mortality neither at 1 year nor 1 month (respectively AUC = 0.51 CI_95_ (0.31–0.72) and AUC = 0.56 CI_95_ (0–0.90)). RVSWI showed modest correlation with both PGD 3‐T72 (AUC = 0.74 CI_95_ (0.49–0.93)) and intraoperative ECMO (AUC = 0.76 CI_95_ (0.64–0.87)). We extracted Youden indexes from the two latter curves that were, respectively, at RVSWI = 13.5 (sensitivity = 0.86 and specificity = 0.68) and RVSWI = 13.8 (sensitivity = 0.53 and specificity = 0.87). Therefore, we selected RVSWI = 13.5 as the threshold for subsequent analyses. Main characteristics of the two groups RVSWI ≤ 13.5 and RVSWI > 13.5 are reported in Table 4.

**TABLE 4:** Recipient, donor and lung transplant characteristics of the right ventricular stroke work index (RVSWI) groups.

Variables	RVSWI ≤ 13.5, *n* = 41	RVSWI > 13.5, *n* = 25	*p* value
Recipient characteristics			
Male	24 (58)	12 (48)	
Body mass index, kg m^−2^	24.0 ± 4.39	22.5 ± 3.67	
Age on transplant day, years	59.0 ± 7.59	54.7 ± 10.3	
Bilateral transplant	36 (88)	24 (96)	
Pulmonary disease			**0.047**
Fibrosis	17 (41)	10 (40)	
Emphysema	20 (49)	6 (24)	
Cystic fibrosis	1 (2)	4 (16)	
Other bronchiectasis	3 (7)	4 (16)	
Other	0 (0)	1 (4)	
Super‐urgent procedure	1 (2)	3 (12)	0.148
ECMO pretransplantation	1 (2)	3 (12)	0.148
Pretransplantation heart functional assessment			
Right‐heart catheterisation			
mPAP, mmHg	27.0 (24.0–31.0)	38.0 (32.0–47.0)	**<** **0.001**
Right atrial pressure, mmHg	7.20 ± 4.09	6.88 ± 4.35	
Cardiac index, L min^−1^ m^−2^	2.6 (2.1–3.2)	3.3 [2.7–3.8]	**0.006**
Pcap, mmHg	9.95 ± 3.94	9.40 ± 4.23	
Pulmonary vascular resistance, Wood unit	4.1 (2.9–5.1)	5.1 (3.6–8.5)	**0.011**
RVSWI, g m m^−2^ beat^−1^	8.7 (6.5–10.9)	16.0 (15.0–18.6)	**<** **0.001**
Echocardiographic right‐heart assessment			
TAPSE < 18 mm	6 (15)	2 (8)	
PAPs estimation on tricuspid regurgitation	44.9 ± 11.0	65.3 ± 19.1	**<** **0.001**
Abnormal right ventricle geometry (hypertrophied, dilated or both)	14 (34)	16 (64)	**0.008**
Magnetic resonance cardiac imaging			
RVEF, %	27.5 ± 10.4	25.3 ± 12.9	
Lung transplantation procedure and outcomes			
Donor characteristics			
Age	50.7 ± 18.1	49.8 ± 17.1	
Sex mismatch	8 (19)	12 (48)	**0.030**
Smoking history	16	10	
Last known donor PaO_2_/FiO_2_ ratio	354 (310–442)	362 (320–464)	
Ex vivo lung perfusion	13 (32)	6 (24)	
First lung ischemia, min	301 (260–514)	298 (248–368)	
Second lung ischemia, min	415 (381–611)	414 (348–568)	
Single lung ischemia, min	302 (274–455)	281 (281–281)	
FiO_2_ first lung reperfusion	0.75 (0.55–1)	0.9 (0.6–1)	
FiO_2_ second lung reperfusion	0.75 (0.52–1)	1 (0.6–1)	
VA ECMO during surgery	16 (39)	20 (80)	**0.003**
VA ECMO during surgery only	9 (22)	9 (36)	
VA ECMO maintained postsurgery	7 (17)	11 (44)	**0.036**
ECMO duration, days	4 (3–6)	5 (3–14)	
Mortality at 30 days	2 (5)	1 (4)	
Mortality at 1 year	6 (15)	6 (25)	
PGD Grade 3 at T72	1 (2)	6 (24)	**0.010**
PGD ungradable at T72	4 (10)	3 (12)	
Posttransplantation heart functional assessment			
Echocardiographic right‐heart assessment			
PAPs estimation on tricuspid regurgitation	27 (25–32)	30 (28–32)	0.30
Abnormal right ventricle geometry (hypertrophied, dilated or both)	1 (2)	2 (8)	0.42

*Note:* Continuous variables are expressed as median (interquartile range 25–75) or as mean ± standard deviation; categorical variables are expressed as No. (%). Bold values represent significative *p* values for exploratory comparisons between the groups RVSWI ≤ 13.5 and RVSWI > 13.5.

Abbreviations: ECMO = extracorporeal membrane oxygenation, mPAP = mean pulmonary artery pressure, PAPs = systolic pulmonary arterial pressure, Pcap = capillary pulmonary pressure, PGD = primary graft dysfunction, RVEF = right ventricular ejection fraction, VA = veno‐arterial.

### 3.3. Outcome Measures

The primary endpoint, 1‐year mortality, did not statistically differ between the two groups: six patients (14.6%) died within the first year in the group RVSWI ≤ 13.5 and six patients (24%) in the group RVSWI > 13.5 (*p* = 0.35) (Figure 2). In the multivariate analysis, RVSWI remained not statistically associated with 1‐year mortality (OR = 0.95 CI_95_(0.71–1.27)). No variable used in our multivariate model was statistically associated with 1‐year mortality (Figure 3). In our study population, 75th percentile for RVSWI was 15.2. We computed a sensitivity analysis exploring the same endpoint with this threshold: it did not show significant association between RVSWI and 1‐year mortality and hence was consistent with the previous analysis (data not shown).

**FIGURE 2:**
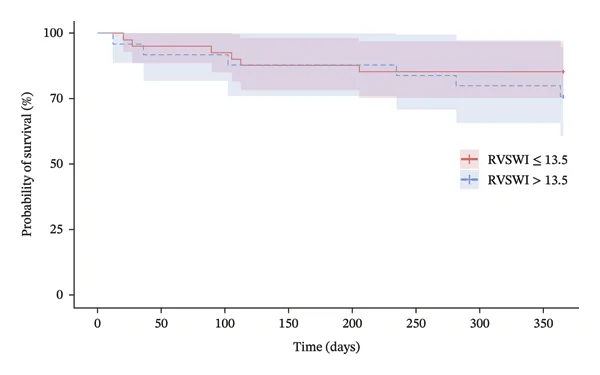
Kaplan–Meier survival curve at 1 year for RVSWI ≤ 13.5 and RVSWI > 13.5.

**FIGURE 3:**
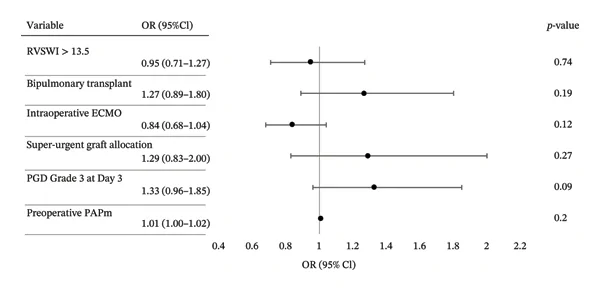
Multivariate analysis for variables associated with 1‐year mortality. Abbreviations: RVSWI = right ventricular stroke work index, ECMO = extracorporeal membrane oxygenation, PGD = primary graft dysfunction, mPAP = mean pulmonary artery pressure.

The secondary endpoints were 1‐month mortality, PGD 3‐T72 and intraoperative ECMO. Mortality at 30 days did not differ between the subgroups. Incidence of PGD 3‐T72 was statistically more frequent in the group RVSWI > 13.5 with 6 patients (24%) achieving the endpoint comparatively with 1 patient (2%) in the group RVSWI ≤ 13.5 (*p* = 0.01). As we previously stated, ROC curve analysis with RVSWI = 13.5 also showed this association with sensitivity = 0.86 and specificity = 0.68. Also, predictive positive value (PPV) was 46% and predictive negative value (PNV) was 94%. Intraoperative ECMO differed between our groups: 20 patients (80%) when RVSWI > 13.5 and 16 patients (39%) when RVSWI ≤ 13.5 (*p* = 0.003). ROC curve analysis with RVSWI = 13.8 had sensitivity = 0.53, specificity = 0.87, PPV = 95% and PNV = 28%.

## 4. Discussion

In a study population of selected lung transplant patients with Group 3 PH, no statistically significant association was observed between RVSWI and 1‐year mortality. However, a higher RVSWI seemed to be associated with intraoperative ECMO use and PGD 3‐T72.

We tried to mitigate the limited statistical power of our study, due to the small number of events of deaths at 1 year and the possible overfitting of our multivariable analysis, by adding a sensitivity analysis which showed concordant results with our logistic regression model and the ROC curve analyses. Nonetheless, strict conclusions cannot be drawn from our results. Because of the small number of events of PGD 3‐T72 in our study, we could not compute further statistical analyses to adjust for potential covariates. Notably, our groups RVSWI ≤ 13.5 and RVSWI > 13.5 mainly differ on two characteristics related to this matter: sex mismatch (more frequently occurring in the RVSWI > 13.5 group) and lung‐disease phenotype (with both groups having around 40% of fibrosis‐related conditions). We graded PGD at different time points as per the guidelines (Table 3 and supporting tables).

In our study, we report a low rate of RHC preoperative assessment with 11% of our cohort while more than 30% had a medical history consistent with PH. Recent guidelines recommend RHC in patients with known PH [21]. Our patients went through right‐heart functional assessment (see supporting tables) but catheterisation was not systematic. RHC is only undertaken when deemed appropriate by the multidisciplinary team assessing patient’s suitability for Ltx, due to its availability and possible complications. Hence, a significant proportion of patients with PH might not have been included. Also, we included patients mostly after January 2020 (45 patients), which is concurrent with SARS‐CoV‐2 pandemic and later with a shift from cystic fibrosis to fibrosis patterns in transplant indication. These changes implied variability in graft availability and recipient’s chronic lung‐disease phenotypes. It probably interfered with the homogeneity of our cohort, specifically not only on preoperative assessment but also on general management.

RVSWI is a surrogate for right ventricular inotropic function: it reflects the workload conditions and the RV ability to overcome them. Its correct units have been subject to recent discussions [22, 23]. In a healthy population, considering approximative standard hemodynamic values (i.e., mPAP = 10–18 mmHg, mRAP = 0–5 mmHg and stroke volume = 60–80 mL), RVSWI should be somewhat ranged between 8 and 10 g m m^−2^ beat^−1^. RVSWI increases reflect both a worsening pulmonary vascular environment and the concomitant RV adaptation, which maintains CI at normal levels. When RVSWI hits unexpectedly low values in patients with various PH aetiologies, outcomes are severely impaired [16, 24, 25]. Armstrong et al. were the first authors to report RVSWI in a Ltx setting and found it to be independently associated with 1‐year mortality when increased [17]. In their cohort, survivors had a lower mean RVSWI (8.99) which compares to our group RVSWI ≤ 13.5.

We found that a high RVSWI was associated with intraoperative ECMO that was mainly implemented before first lung reperfusion, i.e., when the RV is facing acutely increased afterload (pulmonary arterial clamping). Acute right‐heart failure is the leading cause of ECMO implementation in our cohort: a high RVSWI in this setting, reflecting a severe pulmonary vascular disease, might simply be linked with perioperative decision‐making for patients suffering with worse PH without representing per se a valid predictive marker of ECMO use. When considering PGD 3‐T72 higher incidence with high RVSWI, a potential hypothesis could be suggested by some works showing that high transpulmonary flows through the lung graft during the early reperfusion phase could negatively affect the capillary vasculature [26, 27]. In this phase, when RV afterload is acutely reduced, a mismatch could occur between a “high” or “adapted” workload capacity and a new pulmonary vasculature opposing a much lesser resistance, leading to increased pulmonary blood flow responsible for mechanical shear stress lesions on a highly vulnerable capillary endothelium. Lesional pulmonary oedema could occur and impair graft function [28–30]. We think VA ECMO could interfere with this process by unloading transpulmonary workload, but the magnitude of a potential effect is unsure.

Of note, we studied a monocentric population from a high‐volume centre. Hence, our results might lack generalisability. Strategies addressing PH, mechanical support, lung protection strategies or specific diseases such as fibrosis differ from one centre to another. Nevertheless, the concurrence of poor outcomes within different cohorts of Ltx patients when considering high RVSWI might hint an interesting path forward for research.

Further studies are needed to weigh the ability of RVSWI to properly assess the prognosis of patients in the Ltx setting. In our opinion, owing to complex pathophysiology and surgical constraints, a comprehensive approach of right‐heart function and pulmonary circulation might prove beneficial. mPAP and PVR have been extensively studied, but we lack integration of dynamic approaches such as pulmonary vascular compliance and RV–arterial coupling [31, 32]. As a global workload capacity surrogate, and because it can be part of preprocedural assessment, RVSWI might be a useful tool to guide perioperative management. Follow‐up right‐heart assessment could also give valuable insights into the pathophysiology and evolution of RV–arterial coupling. Noticeably, in our study, preoperative right‐heart echocardiographic features (RV geometry and systolic PAP estimation with tricuspid regurgitation) differed between our two groups. When these features were secondarily assessed in stable postoperative conditions, no differences remained.

## 5. Conclusion

In conclusion, no statistically significant association was observed between RVSWI and 1‐year mortality in a retrospective cohort of selected Ltx patients with Group 3 PH. A higher RVSWI was associated with significant procedural outcomes such as ECMO intraoperative use and PGD Grade 3 at Day 3.

## Author Contributions

Yann Marquet, Sébastien Jacqmin, Julien Fessler, Pierre Hammoum and Morgan Le Guen contributed to the study conception and design. Yann Marquet, Sébastien Jacqmin and Julien Fessler contributed to data collection. Pierre Hammoum and Morgan Le Guen computed the statistical analysis. Yann Marquet, Sébastien Jacqmin, Julien Fessler, Pierre Hammoum, Edouard Sage and Morgan Le Guen contributed to the manuscript redaction.

## Funding

The authors did not receive any specific funding for the present work.

## Disclosure

All authors approved the final version of the manuscript.

## Conflicts of Interest

The authors declare no conflicts of interest.

## Supporting Information

Additional supporting information can be found online in the Supporting Information section.

## Supporting information


**Supporting Information** Supporting Table 1. Study flowchart. Supporting Table 2. Population’s variables for other pretransplantation heart functional assessment. Supporting Table 3. Incidence and evolution of PGD grading at different time frames according to RVSWI thresholds.

## Data Availability

The data that support the findings of this study are available from the corresponding author upon reasonable request.
