# Supplementary material for: Right Ventricular Stroke Work Index and Its Prognostic Value in a Lung Transplant Population With Group 3 Pulmonary Hypertension
Source: J Transplant. 2026 Oct 13;2026:1628090. doi: 10.1155/joot/1628090 (PMC13662410; doi:10.1155/joot/1628090)
Supplement: Supplementary file 1 [file JOOT-2026-1628090-s001.zip › STROBE-checklist-RVSWI_PH_lungtransplant.docx]

STROBE Statement—checklist of items that should be included in reports of observational studies

|  | Item No. | Recommendation | Page  No. | Relevant text from manuscript |
| --- | --- | --- | --- | --- |
| **Title and abstract** | 1 | (*a*) Indicate the study’s design with a commonly used term in the title or the abstract | 3 |  |
|  |  | (*b*) Provide in the abstract an informative and balanced summary of what was done and what was found | 3 |  |
| Introduction | | | |  |
| Background/rationale | 2 | Explain the scientific background and rationale for the investigation being reported | 4 | “Low RVSWI values in these settings […] To our knowledge, there are no reports on RVSWI evaluation amongst Ltx recipients with group 3 PH.” |
| Objectives | 3 | State specific objectives, including any prespecified hypotheses | 5 | “. We hypothesised that in our study population, a high RVSWI would lead to an increase in mortality. We further hypothesised that PGD incidence would differ amongst low RVSWI and high RVSWI populations” |
| Methods | | | |  |
| Study design | 4 | Present key elements of study design early in the paper | 5 | We retrospectively investigated adult patients who underwent a lung transplantation in a French tertiary care hospital from January 2013 to December 2023 |
| Setting | 5 | Describe the setting, locations, and relevant dates, including periods of recruitment, exposure, follow-up, and data collection | 5 | Cf. supra |
| Participants | 6 | (*a*) *Cohort study*—Give the eligibility criteria, and the sources and methods of selection of participants. Describe methods of follow-up  *Case-control study*—Give the eligibility criteria, and the sources and methods of case ascertainment and control selection. Give the rationale for the choice of cases and controls  *Cross-sectional study*—Give the eligibility criteria, and the sources and methods of selection of participants | 5 | We assessed for eligibility patients who had a pre-operative history of PH. Patients were included if they went through RHC with haemodynamic assessment less than 12 months before surgery and had a concomitant diagnosis of pre-capillary or combined pre-and post-capillary PH. We did not include patients who underwent multi-organ transplantation (*i.e.* other organ transplantation on the same surgical procedure), redo transplant procedure or when cardiopulmonary bypass was used during surgery. Patients who lacked data to properly assess mortality up to 1 year and PGD grading were excluded. Patients who lacked data to properly assess mortality up to 1 year and PGD grading were excluded |
|  |  | (*b*) *Cohort study*—For matched studies, give matching criteria and number of exposed and unexposed  *Case-control study*—For matched studies, give matching criteria and the number of controls per case |  |  |
| Variables | 7 | Clearly define all outcomes, exposures, predictors, potential confounders, and effect modifiers. Give diagnostic criteria, if applicable | 5-8 |  |
| Data sources/ measurement | 8* | For each variable of interest, give sources of data and details of methods of assessment (measurement). Describe comparability of assessment methods if there is more than one group | 5-8 |  |
| Bias | 9 | Describe any efforts to address potential sources of bias | 8 | The primary endpoint was assessed using logistic regression analysis. Initially, a bivariate analysis was conducted to explore associations between clinical variables and the primary outcome. Variables with clinical relevance or statistical significance in bivariate analysis were included in a multivariate logistic regression model adjusted for key baseline covariates associated with early mortality in the literature, including double-lung transplant, ECMO use during surgery, HELT procedure, PGD 3-T72, and mPAP  +  A sensitivity analysis of the primary endpoint was performed using the same methodology, applying an alternative RVSWI threshold defined as the 75th percentile of RVSWI in our study population. |
| Study size | 10 | Explain how the study size was arrived at | N/A |  |

Continued on next page

| Quantitative variables | 11 | Explain how quantitative variables were handled in the analyses. If applicable, describe which groupings were chosen and why | 7 | Continuous variables, expressed as mean (± standard deviation) or median [25th–75th percentile interquartile range (IQR)], were compared using Student’s t-test or Wilcoxon’s rank-sum test, depending on data distribution. The distribution of continuous variables was evaluated using graphical data visualization techniques. |
| --- | --- | --- | --- | --- |
| Statistical methods | 12 | (*a*) Describe all statistical methods, including those used to control for confounding | 7-8 | *Outcome measures, data assessment and statistical analyses* |
|  |  | (*b*) Describe any methods used to examine subgroups and interactions | N/A |  |
|  |  | (*c*) Explain how missing data were addressed | N/A |  |
|  |  | (*d*) *Cohort study*—If applicable, explain how loss to follow-up was addressed  *Case-control study*—If applicable, explain how matching of cases and controls was addressed  *Cross-sectional study*—If applicable, describe analytical methods taking account of sampling strategy | N/A |  |
|  |  | (*e*) Describe any sensitivity analyses | 8 | Cf. supra |
| Results | | | | |
| Participants | 13* | (a) Report numbers of individuals at each stage of study—eg numbers potentially eligible, examined for eligibility, confirmed eligible, included in the study, completing follow-up, and analysed | 8 |  |
|  |  | (b) Give reasons for non-participation at each stage | 8 |  |
|  |  | (c) Consider use of a flow diagram | Supplementary material | Supplementary 1 |
| Descriptive data | 14* | (a) Give characteristics of study participants (eg demographic, clinical, social) and information on exposures and potential confounders | 8-9 | Table 1 |
|  |  | (b) Indicate number of participants with missing data for each variable of interest | N/A |  |
|  |  | (c) *Cohort study*—Summarise follow-up time (eg, average and total amount) | N/A |  |
| Outcome data | 15* | *Cohort study*—Report numbers of outcome events or summary measures over time | N/A |  |
|  |  | *Case-control study—*Report numbers in each exposure category, or summary measures of exposure |  |  |
|  |  | *Cross-sectional study—*Report numbers of outcome events or summary measures |  |  |
| Main results | 16 | (*a*) Give unadjusted estimates and, if applicable, confounder-adjusted estimates and their precision (eg, 95% confidence interval). Make clear which confounders were adjusted for and why they were included | 10 |  |
|  |  | (*b*) Report category boundaries when continuous variables were categorized | 10 |  |
|  |  | (*c*) If relevant, consider translating estimates of relative risk into absolute risk for a meaningful time period |  |  |

Continued on next page

| Other analyses | 17 | Report other analyses done—eg analyses of subgroups and interactions, and sensitivity analyses | 10 |  |
| --- | --- | --- | --- | --- |
| Discussion | | | | |
| Key results | 18 | Summarise key results with reference to study objectives | 11 |  |
| Limitations | 19 | Discuss limitations of the study, taking into account sources of potential bias or imprecision. Discuss both direction and magnitude of any potential bias | 11-12 |  |
| Interpretation | 20 | Give a cautious overall interpretation of results considering objectives, limitations, multiplicity of analyses, results from similar studies, and other relevant evidence | 12 |  |
| Generalisability | 21 | Discuss the generalisability (external validity) of the study results | 12 |  |
| Other information | |  | | |
| Funding | 22 | Give the source of funding and the role of the funders for the present study and, if applicable, for the original study on which the present article is based | 14 |  |

*Give information separately for cases and controls in case-control studies and, if applicable, for exposed and unexposed groups in cohort and cross-sectional studies.

**Note:** An Explanation and Elaboration article discusses each checklist item and gives methodological background and published examples of transparent reporting. The STROBE checklist is best used in conjunction with this article (freely available on the Web sites of PLoS Medicine at http://www.plosmedicine.org/, Annals of Internal Medicine at http://www.annals.org/, and Epidemiology at http://www.epidem.com/). Information on the STROBE Initiative is available at www.strobe-statement.org.
