# Supplementary material for: Right Ventricular Stroke Work Index and Its Prognostic Value in a Lung Transplant Population With Group 3 Pulmonary Hypertension
Source: J Transplant. 2026 Oct 13;2026:1628090. doi: 10.1155/joot/1628090 (PMC13662410; doi:10.1155/joot/1628090)
Supplement: Supplementary file 1 [file JOOT-2026-1628090-s001.zip › RVSWI_supplementary_1.docx]

**SUPPLEMENTARY TABLES**

**Supplementary Table 1. Flow-chart of the study**

 **CAPTION :** PH = pulmonary hypertension, CPB = cardiopulmonary bypass
