# Supplementary material for: Right Ventricular Stroke Work Index and Its Prognostic Value in a Lung Transplant Population With Group 3 Pulmonary Hypertension
Source: J Transplant. 2026 Oct 13;2026:1628090. doi: 10.1155/joot/1628090 (PMC13662410; doi:10.1155/joot/1628090)
Supplement: Supplementary file 1 [file JOOT-2026-1628090-s001.zip › RVSWI_supplementary_2.docx]

| **Supplementary Table 2. Other pre-transplantation heart functional assessment** | |
| --- | --- |
| **Variables** |  |
| **Pre-operative echocardiography** | **n = 65** |
| Left ventricular systolic function |  |
| Normal EF | 63 (97) |
| Mildly reduced EF | 2 (3) |
| Reduced EF | 0 (0) |
| Left ventricular diastolic function assessed through transmitral flow |  |
| Relaxation impairment | 25 (38) |
| Restrictive pattern | 0 (0) |
| Valvulopathy | 0 (0) |
| Right Ventricle geometry |  |
| Normal | 35 (54) |
| Isolated dilatation | 23 (35) |
| Isolated hypertrophy | 1 (2) |
| Dilatation and hypertrophy | 6 (9) |
| TAPSE <18mm | 8 (12) |
| Right Atrium geometry |  |
| Right atrial dilatation | 23 (35) |
| PAPs estimation on tricuspid regurgitation | 49 [40-63] |
| **Magnetic resonance cardiac imaging** | **n = 8** |
| LVEF, % | 50 [49-56] |
| RVEF, % | 20 [19-36] |
| Abnormal RV late-gadolinium enhancement or edema | 0 (0) |
| **Post-anesthesia induction Swan-Ganz on transplantation procedure** | **n = 62** |
| Swan Ganz measurements obtained without ECMO assistance | n = 58 |
| PAPs, mmHg | 48 [40-58] |
| PAPd, mmHg | 30 [22-37] |
| PAPm, mmHg | 37 [30-46] |
| Cardiac index, L.min^-1^.m^-2^ | 2.7 [2.3-3.2] |
| Abbreviations: EF = ejection fraction, TAPSE = Tricuspid Annular Plane Systolic Excursion, LVEF = left ventricle ejection fraction, RVEF = Right ventricle ejection fraction, PAPs = systolic pulmonary artery pressure, PAPd = diastolic pulmonary artery pressure, PAPm = mean pulmonary artery pressure.  Continuous variables are expressed as median [interquartile range 25-75]; categorical variables are expressed as No. (%). | |
