# Supplementary material for: Right Ventricular Stroke Work Index and Its Prognostic Value in a Lung Transplant Population With Group 3 Pulmonary Hypertension
Source: J Transplant. 2026 Oct 13;2026:1628090. doi: 10.1155/joot/1628090 (PMC13662410; doi:10.1155/joot/1628090)
Supplement: Supplementary file 1 [file JOOT-2026-1628090-s001.zip › RVSWI_supplementary_3.docx]

**Supplementary Table 3. Incidence and evolution of PGD grading at different time frames according to RVSWI thresholds**
